# A diagnostic methodology for Alzheimer’s disease

**DOI:** 10.1186/2043-9113-3-9

**Published:** 2013-04-25

**Authors:** Wen-Chin Hsu, Christopher Denq, Su-Shing Chen

**Affiliations:** 1 Systems Biology Lab, University of Florida, Gainesville, FL, 32611, USA; 2 Department of Electrical and Computer Engineering, University of Florida, Gainesville, FL, 32611, USA; 3 Van Nuys Senior High School, Van Nuys, CA, 91411, USA; 4 Department of Computer and Information Science and Engineering, University of Florida, Gainesville, FL, 32611, USA

**Keywords:** Feature selection, Biomarkers, Target networks, Alzheimer’s disease, Support vector machine

## Abstract

**Background:**

Like all other neurodegenerative diseases, Alzheimer’s disease (AD) remains a very challenging and difficult problem for diagnosis and therapy. For many years, only historical, behavioral and psychiatric measures have been available to AD cases. Recently, a definitive diagnostic framework, using biomarkers and imaging, has been proposed. In this paper, we propose a promising diagnostic methodology for the framework.

**Methods:**

In a previous paper, we developed an efficient SVM (Support Vector Machine) based method, which we have now applied to discover important biomarkers and target networks which provide strategies for AD therapy.

**Results:**

The methodology selects a number of blood-based biomarkers (fewer than 10% of initial numbers on three AD datasets from NCBI), and the results are statistically verified by cross-validation. The resulting SVM is a classifier of AD vs. normal subjects. We construct target networks of AD based on MI (mutual information). In addition, a hierarchical clustering is applied on the initial data and clustered genes are visualized in a heatmap. The proposed method also performs gender analysis by classifying subjects based on gender.

**Conclusions:**

Unlike other traditional statistical analyses, our method uses a machine learning-based algorithm. Our method selects a small set of important biomarkers for AD, differentiates noisy (irrelevant) from relevant biomarkers and also provides the target networks of the selected biomarkers, which will be useful for diagnosis and therapeutic design. Finally, based on the gender analysis, we observe that gender could play a role in AD diagnosis.

## Background

### Overview

Analysis of Alzheimer’s disease (AD) has been a challenging problem for diagnosis and therapy. Currently, a definitive clinical diagnosis can be obtained only by historical, behavioral and psychiatric measures, and only when the patient’s condition has sufficiently deteriorated [1]. In [2], a dynamic model was proposed for AD diagnosis and has led to several studies of biomarker-based analysis. However, in order to validate the model, continuous studies of biomarkers are necessary to identify critical time points when changes or permutations of biomarkers occur [3]. The specificity and sensitivity of AD diagnosis remain in doubt due to the lack of comparisons of AD with other neurodegenerative disease [4]. In addition, the standards and guidelines for blood-sample biomarkers are still in the process of development [4]. The current methods for biomarker collection are also problematic, due to the need for expensive instrumentation and the invasiveness of the procedures [5]. Recently, Zhang et al. integrated three modalities--MRI, FDG-PET, and CSF biomarker--into a Multi-Kernel SVM to classify AD vs. normal samples [6].

### NIH guidelines

In the past few years, the technologies for both biomarker analysis and imaging have provided promising contributions to definitive diagnoses of AD. In 2011, the NIH National Institute on Aging and the Alzheimer’s Association also established new guidelines to allow use of biomarkers and imaging for diagnoses [1]. Since the announcement from NIH, research to identify and compare biomarkers has been thriving.

### SVM

High-dimensional pattern classifiers such as SVMs (Support Vector Machines) are adapted to contribute classifications. In [7-9], biomarker selections were performed by SVM-RFE, a feature (biomarker) extraction method, by reclusively eliminating features based on the validation accuracy of SVM [10]. During the selection process, the least useful feature is iteratively removed from the original subset. However, a group of weak features can still construct a strong classifier [10]. Once a feature is removed from the original subset, it cannot be evaluated by different combinations of the remaining features. Thus, the SVM-RFE approach usually suffers from selection of a sub-optimal subset since the classification ability of features should be evaluated by subsets instead of by individuals.

### AMFES

In [11], we first proposed an efficient algorithm, AMFES (Adaptive Multiple FEatues Selection), to select important biomarkers for cancers. Based on that initial success, this paper reports the extension of previous results on the datasets provided by Maes et al. in an attempt to discover important biomarkers for AD from the blood-based samples [12]. Unlike traditional statistical analyses, AMFES is an SVM-based methodology, which can select a much smaller subset of important biomarkers. In addition, AMFES applies an adaptive method which enables selection of a globally optimal subset of important biomarkers compared to SVM-RFE. AMFES is particularly useful for differentiating noisy biomarkers from the relevant ones when interferences between biomarkers exist. Our results are supported by a high ROC/AUC (Receiver Operating Characteristic/Area Under Curve) value when we apply a cross-validation verification. Thus, AMFES should play an important role in the classification framework of multi-modalities proposed by Zhang et al. in [6]. In this paper, we shall develop the details of AMFES for blood-based biomarkers.

The target networks of AD with statistical dependencies (mutual information) are constructed by these selected biomarkers. The resulting AD target network is characterized as a signature of the disease, and will enable a more detailed diagnosis. In addition, the MI (Mutual Information) values of AD subjects are found to be lower than those of normal subjects. Based on our method and results, a promising framework for definitive diagnosis is proposed.

The organization of this paper is as follows: The Methods section describes AMFES [11], as well as the computations of mutual information between two biomarkers and the construction of the target networks. In the Results section, we describe the PBMC (Peripheral Blood Mononuclear Cells) datasets of sporadic AD: GSE4226, GSE4227, and GSE4229 [12-14]. In addition, we describe the biomarkers and target networks of AD selected according to our approach on these 3 datasets.

## Methods

### AMFES

Selecting a small subset out of hundreds and thousands of features is always a challenging task due to the COD (Curse of Dimensionality) problem for microarray datasets. To tackle this problem, we use a gene selection methodology, AMFES, to select an optimal subset of genes by training an SVM with subsets of genes generated adaptively [11]. AMFES is developed based on two fundamental processes, ranking and selection.

The gene ranking process contains several stages. In the first stage, all genes are ranked by their ranking scores in a descending order. Then, in the next stage, only the top half of the ranked genes are ranked again, while the bottom half holds the current order in the subsequent stage. The same iteration repeats recursively until only three genes remain to be ranked again to complete one ranking process.

Assume at a given ranking stage, there are *k* genes indexed from *1* to *k*. To rank these *k* genes, we follow 4 steps below. (I) We first generate *m* independent subsets S_*1*_… S_*m*._ Each subset S_*i*_, *i* = 1, 2… *m*, has *j* genes which are selected randomly and independently from the *k* genes (II) Let C_*i*_ be the SVM classifier that is trained on each subset of genes_*,*_*i* = 1, 2… *m*. For each gene of *k* genes, we compute the ranking score *θ*_*m*_(g) of the gene g, as equation (1) below [11]. (III) We use the average weight of gene *g*, given by the summation of weights of *g* in *m* subsets divided by the number of subsets for which *g* is randomly selected. The *weight*_*i*_(g) is then defined as the change in the objective function due to *g* as equation (2) [11] and the *m* value is obtained when θ_m_ satisfies the equation (3) in [11]. This increases the robustness to represent the true classifying ability of gene *g*. (IV) The *k* genes are then ranked in descending order by their ranking scores.

(1) $$\theta_{m}\left(g\right)=\frac{\sum_{i=1}^{m}I_{\left\{g\;\in S_{i}\right\}}{\text{weight}}_{i}\left(g\right)}{\sum_{i-1}^{m}I_{\left\{g\;\in S_{i}\right\}}}$$

where I is an indicator function, such that I_proposition_ = 1 if the proposition is true; otherwise, I_proposition_ = 0. In other words, if gene g is randomly selected for the subset S_i_, it is denoted as gϵS_i_ and I_proposition_ = 1.

We denote the objective function of C_*i*_ as *obj*_*i*_(*v*_1_, *v*_2_, …,*v*_5_) where **v**_1_, **v**_2_… **v**_s_ are support vectors of C_*i*_. The *weight*_*i*_(g) is then defined as the change in the objective function due to g, i.e., [6-8].

(2) $${\mathrm{weight}}_{i}\left(g\right)=\left|\mathrm{ob}j_{i}\left(v_{1},v_{2},\ldots v_{s}\right)-\mathrm{ob}j_{i}\left(v_{1}^{\left(g\right)},v_{2}^{\left(g\right)},\ldots ,v_{3}^{\left(g\right)}\right)\right|$$

Note that if **v** is a vector, **v**^(*g*)^ is the vector obtained by dropping gene g from **v**. Let θ_m_ be a vector comprising the ranking scores derived from the *m* gene subsets generated thus far and θ_m-1_ be the vector at the previous stage. The *m* value is determined when θ_m_ satisfies equation (3) by adding a gene to an empty subset once a time.

(3) $$\frac{{∥\theta_{m-1}\;-\;\theta_{m}∥}^{2}}{{∥\theta_{m-1}∥}^{2}}<\;0.01$$

where ||θ|| is understood as the Euclidean norm of vector θ.

The ranking process is performed by ranking both artificial and original features together. The use of artificial features has been demonstrated as a useful tool to distinguish the relevant features from the irrelevant ones, as in [15-17]. When a set of genes is given, we generate artificial genes and rank them together with original ones. After finishing the ranking of the set, we assign a gene-index to each original gene by the proportion of artificial ones that are ranked above it, where the gene-index is a real numerical value between 0 and 1. Then, we generate a few subset candidates from which the optimal subset is chosen. Each subset has a subset value, *p*_*i*_*,* and it contains original genes whose indices are smaller than or equal to *p*_*i*_[11]. We train an SVM on every subset, and compute its validation accuracy *v*(*p*_*i*_). We stop at the first *p*_*k*_ at which its validation accuracy is better than baseline (i.e., the case in which all features are involved in training [11]).

When starting to apply AMFES, we first divide all samples into either learning samples or testing samples. Then, we randomly extract *r* training-validation pairs from the learning samples according to the heuristic rule $r=\max\left(5,\left(\mathrm{int}\right)\frac{500}{n+0.5}\right)$, where *n* is the number of learning samples in the dataset. The heuristic ratio and rule are chosen based on experience of the balance of time consumption and performance. The ranking and selection processes from previous sections correspond to one training-validation pair. To increase the reliability of validation, we generate *r* pairs to find the optimal subset.

We calculate the validation accuracy of all pairs and the average accuracy, *av*(*p*_*i*_). Then, we perform the subset search as explained in the previous section to find the optimal *p*_*i*_ value, denoted as p*. However, p* is a derived value and does not belong to a unique subset. Thus, we have to adapt all training samples and repeat the entire process in order to find a unique subset.

We generate artificial genes and rank them together with the original genes. Finally, we select the original genes whose indices are smaller than or equal to the p* derived previously as the subset of genes we select for the dataset [11].

### Mutual information

To treat a complex disease or injury such as AD, an optimal approach is to discover important biomarkers for which we can find certain treatments. These biomarkers form a certain dependency network as a framework for diagnosis and therapy [18]. We call such a network a target network of these biomarkers [11].

Mutual information has been used to measure the dependency between two random variables. Assume the two random variables X and Y are continuous numbers. The mutual information is defined as [19]:

(7) $$I\left(X,Y\right)=\iint f\left(x,y\right)\text{log}\left(\frac{f\left(x,y\right)}{f\left(x\right)f\left(y\right)}\right)\text{dxdy}$$

where *f*(x,y) denotes the joint probability distribution, and *f*(x) and *f*(y) denote the marginal probability distributions of X and Y. By using the Gaussian kernel estimation, the *f*(x, y),*f*(x) and *f*(y) can be further represented as [20]:

(8) $$f\left(x,y\right)=\frac{1}{M}\sum_{2\pi h^{2}}e^{-\;\frac{1}{2h^{2}}\left({\left(x-x_{u}\right)}^{2}+\left(y-y_{u}^{2}\right)\right)}$$

(9) $$f\left(x\right)=\frac{1}{M}\sum \frac{1}{\sqrt{2\pi h^{2}}}e^{\frac{1}{2h^{2}}{\left(x-x_{u}\right)}^{2}}$$

(10) $$f\left(y\right)=\frac{1}{M}\sum \frac{1}{\sqrt{2\pi h^{2}}}e^{\frac{1}{2h^{2}}{\left(y-y_{u}\right)}^{2}}$$

where *M* represents the number of samples for both X and Y, *u* is index of samples *u* = 1,2,…*M*, and *h* is a parameter controlling the width of the kernels. Thus, the mutual information *I*(*X,Y*) can then be represented as:

(11) $$I\left(X,Y\right)=\frac{1}{M}\sum_{i}\log\frac{M\sum_{i}e^{-\frac{1}{2h^{2}}\left({\left(x_{w}-x_{u}\right)}^{2}+{\left(y_{w}-y_{u}\right)}^{2}\right)}}{\sum_{j}e^{-\frac{1}{2h^{2}}{\left(x_{w}-x_{u}\right)}^{2}}\sum_{j}e^{-\frac{1}{2h^{2}}{\left(y_{w}-y_{u}\right)}^{2}}}$$

where both *w, u* are indices of samples *w*,*u* = 1,2,…*M.*

Computation of pairwise genes of a microarray dataset usually involves a nested loops calculation which takes a dramatic amount of time. Assume a dataset has *N* genes and each gene has *M* samples. To calculate the pairwise mutual information values, the computation usually first finds the kernel distance between any two samples for a given gene. Then, the same process goes through every pair of genes in the dataset. In order to be computationally efficient, two improvements are applied [21]. The first one is to calculate the marginal probability of each gene in advance and use it repeatedly during the process [21,22]. The second improvement is to move the summation of each sample pair for a given gene to the most outer for-loop rather than inside a nested for-loop for every pairwise gene. As a result, the kernel distance between two samples is only calculated twice instead *N* times, thereby saving considerable computational time. LNO (Loops Nest Optimization) which changes the order of nested loops is a common time-saving technique in computer science field [23].

### Target network

In our approach, a constructed target network is represented by an undirected graph in which nodes represent genes in the system and edges represent the dependency between gene pairs [18]. For each gene pair, we use MI (Mutual Information) to measure the dependency between them and represent the weight of linkages. Assuming that the graph contains N nodes (genes), there should be $\frac{N*\left(N-1\right)}{2}$ pairwise MI values for all genetic pairs. An adjacency matrix of N × N elements is used to hold MI values of all the linkages in the graph. The adjacency matrix can be visualized as a heatmap. In addition, hierarchical clustering is often used to help verify the dependency between genes. In this paper, we adapt the Matlab clustergram() function, which uses Euclidean distance as the default method to calculate pairwise distance to visualize the heatmap after clustering.

**Figure 1:**
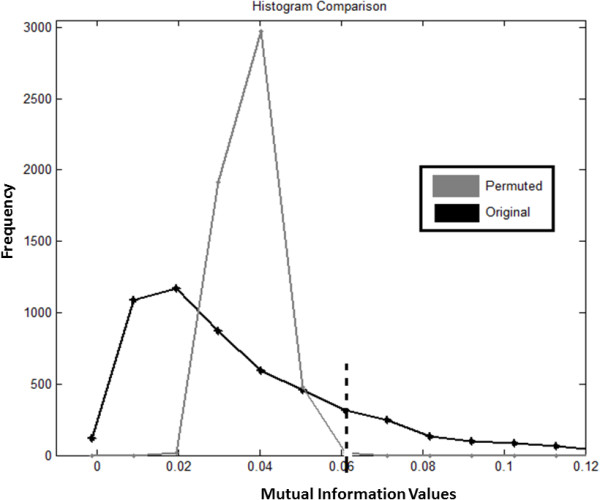
Distributions of original pair-wise MI values and permuted pair-wise MI values.

In order to remove irrelevant linkages in a graph, it is necessary to choose a suitable MI threshold which determines the topology of networks formed. The value of 0 or 1 is assigned to a matrix element based on the chosen MI threshold. References in [24] and [25] describe a method to determine a suitable threshold using permutations of MI. The procedure involves permuting MI values of gene pairs and then choosing the largest MI to be the threshold. Using this procedure for 30 repetitions of the permutation on the MI matrix, we choose 0.06 as the threshold. The distributions of the original and permuted MI values are shown in Figure 1.

## Results

### Microarray datasets descriptions

The gene expressions used for this paper are based on PBMC (Peripheral Blood Mononuclear Cells) blood-based biomarkers [12-14]. Subject AD and normal elderly patients all took the MMSE (Mini-Mental State Examination). Those with chronic metabolic conditions such as diabetes, rheumatoid arthritis and other chronic illnesses or familial AD problems are not included in the analysis [12-14]. Fields such as immunology, transplant immunology, vaccine development often use PBMCs.

### GSE4226

AMFES is used to analyze the gene expressions from the BMC (Blood Mononuclear Cell) of AD patients [12]. The dataset contains 9600 features from 14 normal elderly control samples (7 females and 7 male) and 14 AD patient samples (7 females and 7 males). The average age of the patients is 79 ± 5 years with 11 ± 4 years of formal educational background. The platform of the dataset is GPL1211 and gene expressions are extracted by using the technology of NIA (National Institution on Aging) Human MGC (Mammalian Genome Collection) cDNA microarray. The raw normalized dataset can be found in Additional file 1.

### GSE4227

The dataset GSE4227 was extracted from BMC and under the same GPL1211 platform as GSE4226. It was used to identify the genes with expressions associated with GSTM3 (Glutathione S-Transferase Mu 3) [14]. The dataset contains 9600 features and 34 samples (16 sporadic AD samples and 18 normal elderly control samples). The raw normalized dataset can be found in Additional file 2.

### GSE4229

This dataset contains new subjects and some subjects from GSE4226 and GSE4227 [13]. The blood samples were extracted by phlebotomy in an EDTA vacutainer. The dataset also contains 9600 features and 40 samples (18 AD samples and 22 normal elderly control samples). The platform is the same as GSE4226 and GES4227. The raw normalized dataset can be found in Additional file 3.

### Results of AMFES

Table 1 contains the descriptions of the three datasets, GSE 4226, 4227 and 4229. AMFES selected 74 genes for GSE4226, 52 for GSE4227, and 395 for GSE4229 and the selected results are shown in Table 2. The complete lists of the 74, 52 and 395 selected genes can be found in Additional file 4: GSE4226_74_Biomakrers.xlsx, Additional file 5: GSE4227_52_Biomakrers.xlsx and Additional file 6: GSE4229_395_Biomakrers.xlsx. The statistical results of MI values are shown in Table 3.

**Table 1:** Descriptions of 3 datasets: GSE4226, GSE4227, and GSE4229

	**GSE4226**	**GSE4227**	**GSE4229**
Number of Biomarkers	9600	9600	9600
Type of Biomarkers	RNAs	RNAs	RNAs
Number of Samples	28 (14 AD vs. 14 Normal)	34(14 AD vs. 18 normal)	40(18 AD vs. 22 normal)

**Table 2:** Results of selected subsets of genes

	**GES4226**	**GSE4227**	**GSE4229**
Number of Biomarkers Selected	74	52	395

**Table 3:** Results of analysis of MI matrices

	**Mean value of MI**	**Standard deviation of MI**	**Num of positive values**	**Num of negative values**	**Min value**	**Max value**
GSE4226_normal	0.0245	0.0793	4636	840	−0.0037	1.4451
GSE4226_AD	0.0225	0.0804	4382	1094	−0.0032	1.7946
GSE4227_normal	0.0086	0.0487	2086	618	−0.0029	1.5557
GSE4227_AD	0.0076	0.0466	1972	732	−0.0035	1.4860
GSE4229_normal	0.000095	0.0045	100275	55750	−0.0041	1.1717
GSE4229_AD	0.000081	0.0045	97987	58038	−0.0036	1.5503

### ROC/AUC analysis

**Figure 2:**
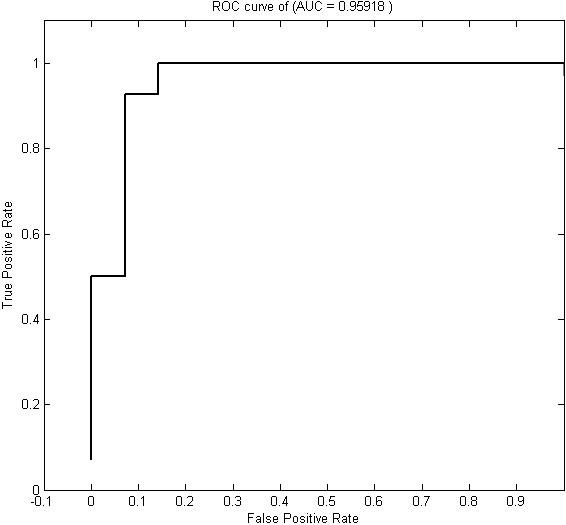
ROC curve of selected genes of GSE4226, 74 genes.

To show the classification ability of selected genes, we calculate the AUC (Area Under the Curve) and draw the ROC (Receiver Operating Characteristic) curves for the expressions of 74 genes by using the LIBSVM Matlab ROC tool, as shown in Figure 2[26]. The ROC/AUC value is verified based on cross-validation [26]. The AUC metric represents the probability that the classifier constructed by the selected genes has higher performance than the classifier constructed using randomly chosen genes. Thus, the closeness of the AUC value to the value of 1 indicates the importance of the selected genes. The ROC/AUC value (0.95918) therefore supports the significance of verification for the selected genes.

### Mutual information analysis

**Figure 3:**
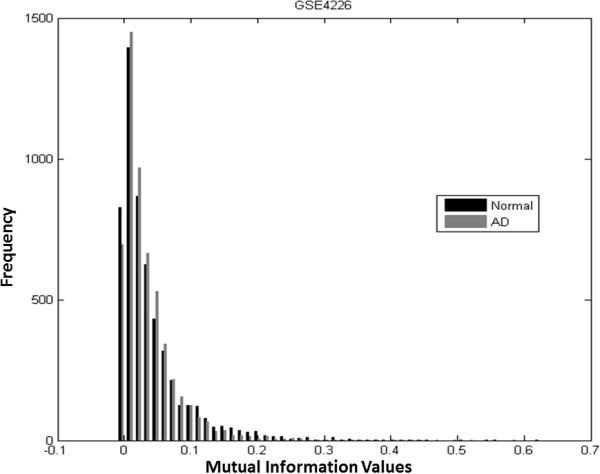
Histograms of pairwise MI values of normal and AD samples of GSE4226.

**Figure 4:**
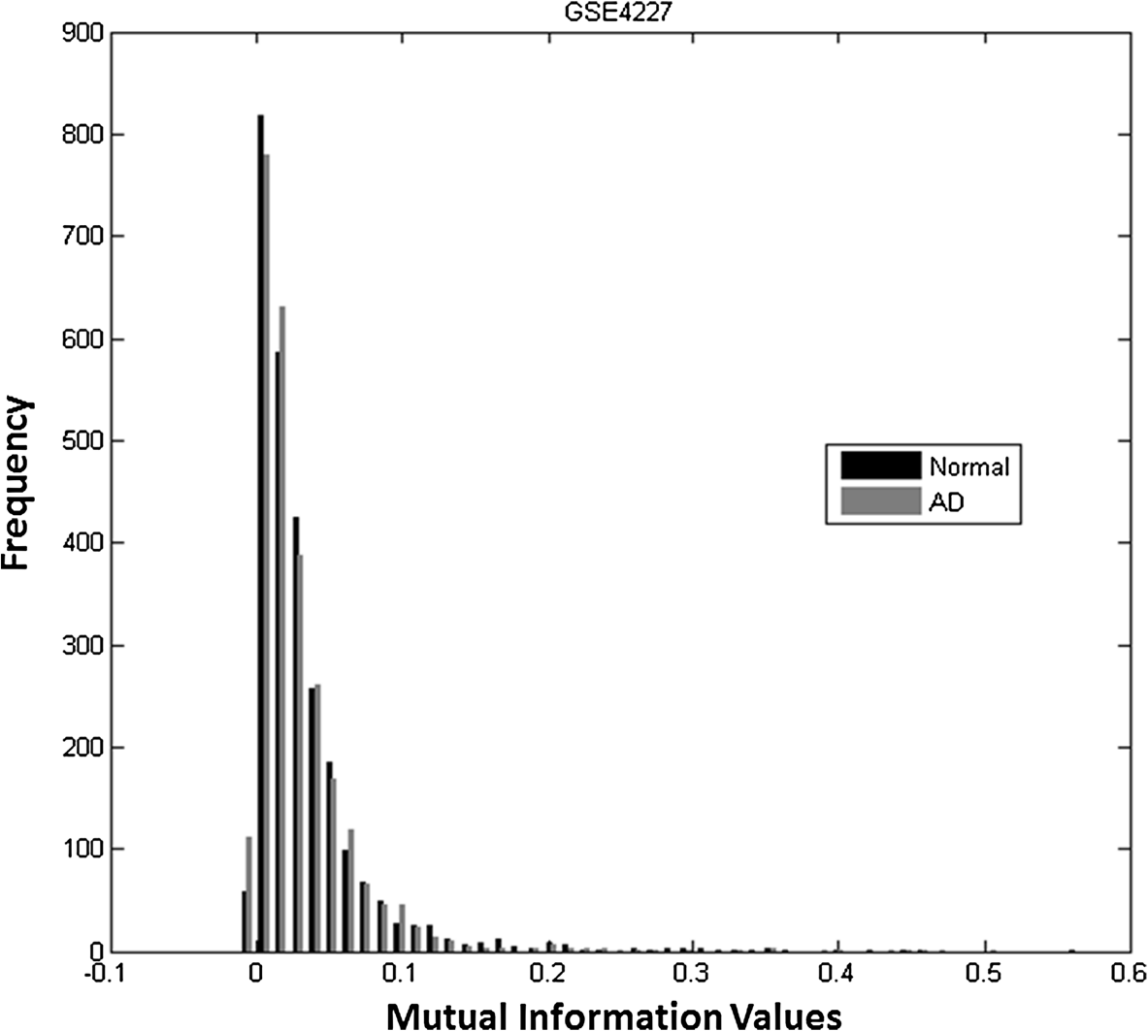
Histograms of pairwise MI values of normal and AD samples of GSE4227.

**Figure 5:**
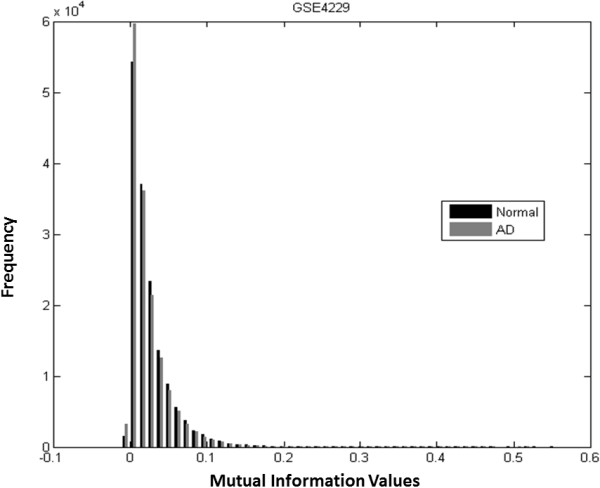
Histograms of pairwise MI values of normal and AD samples of GSE4229.

The pair-wise MI values of selected genes of AD or normal samples are calculated separately. The histograms of MI values of GSE4226 are shown in Figure 3, where the black bars represent MI values of normal samples, and the grey bars are for AD samples. The histograms for GSE4227 and GSE4229 are displayed in Figures 4 and 5, respectively. The pair-wise MI files of AD and normal samples are shown in Additional file 7: GSE4226 AD MI.xlsx, Additional file 8: GSE4226-Normal MI.xlsx, Additional file 9: GSE4227-AD MI.xlsx, Additional file 10: GSE4227-Normal MI.xlsx, Additional file 11: GSE4229-AD MI.xlsx and Additional file 12: GSE4229-Normal MI.xlsx. The analysis results are shown in Table 3. Interestingly, the mean MI value of the GSE4226 AD samples is larger than those of the other datasets. In addition, the ratio of the number of positive MI values to the negative MI values for GSE4226 AD samples is also larger than the ratio for other datasets.

### Clustergram/Target network examples

**Figure 6:**
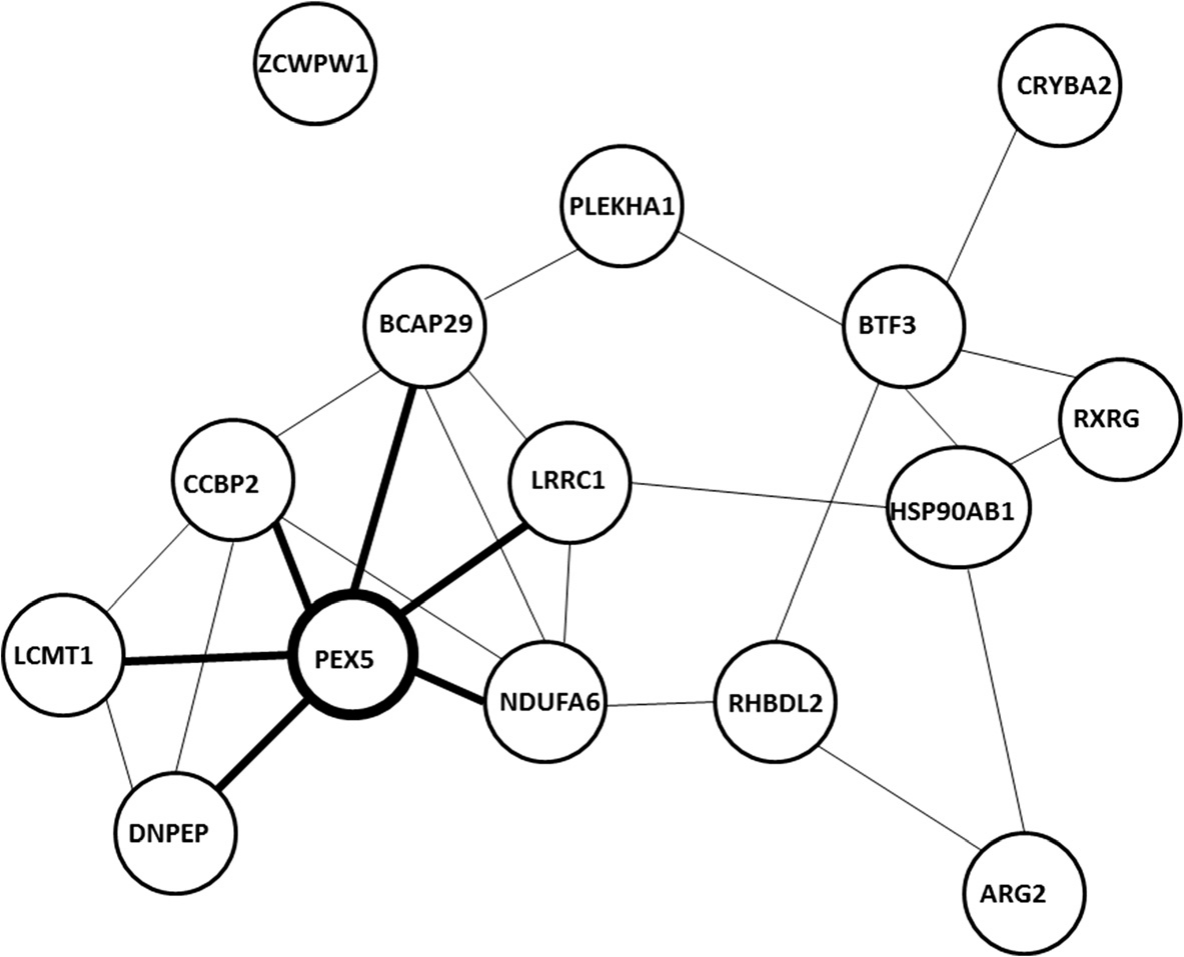
A target network of top ranked 15 genes selected by AMFES for GSE4226.

**Figure 7:**
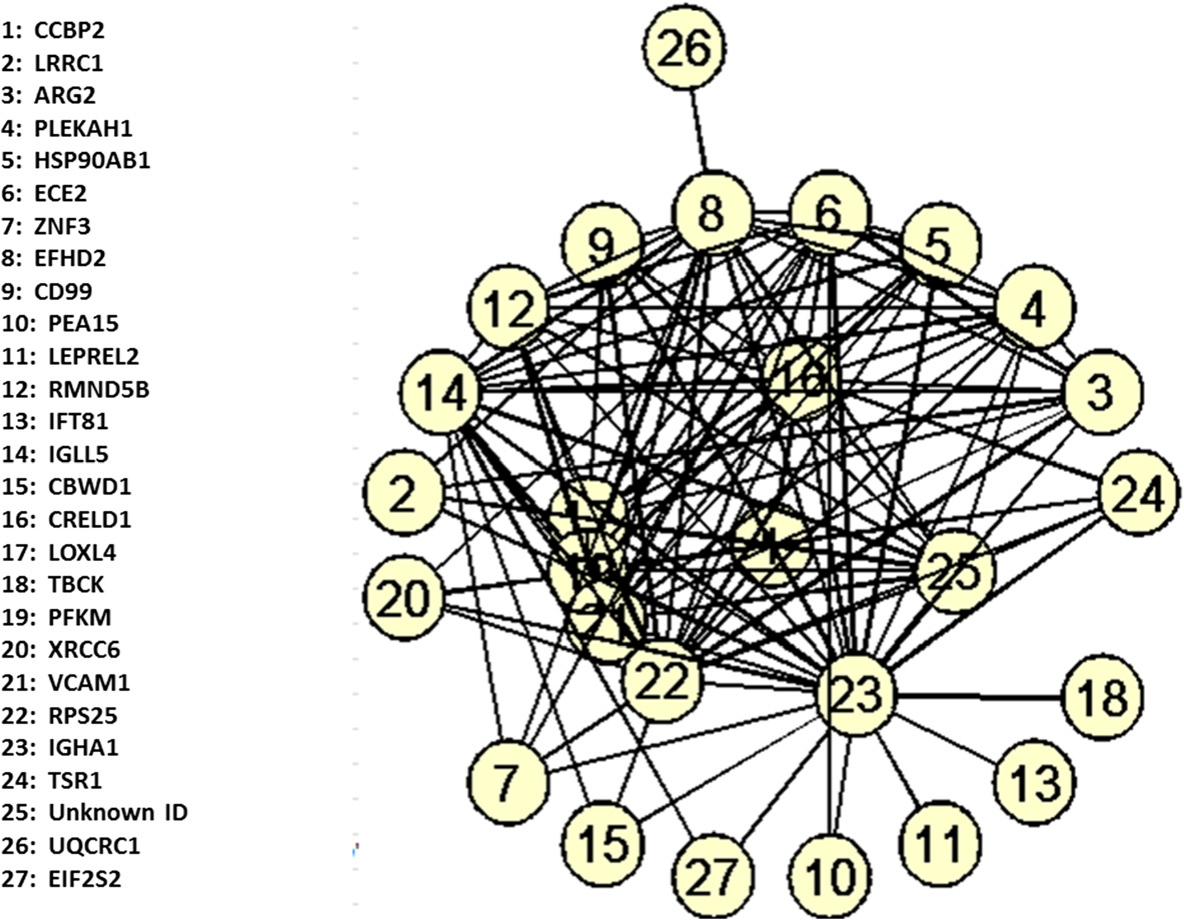
A target network of 74 genes selected by AMFES for GSE4226.

**Figure 8:**
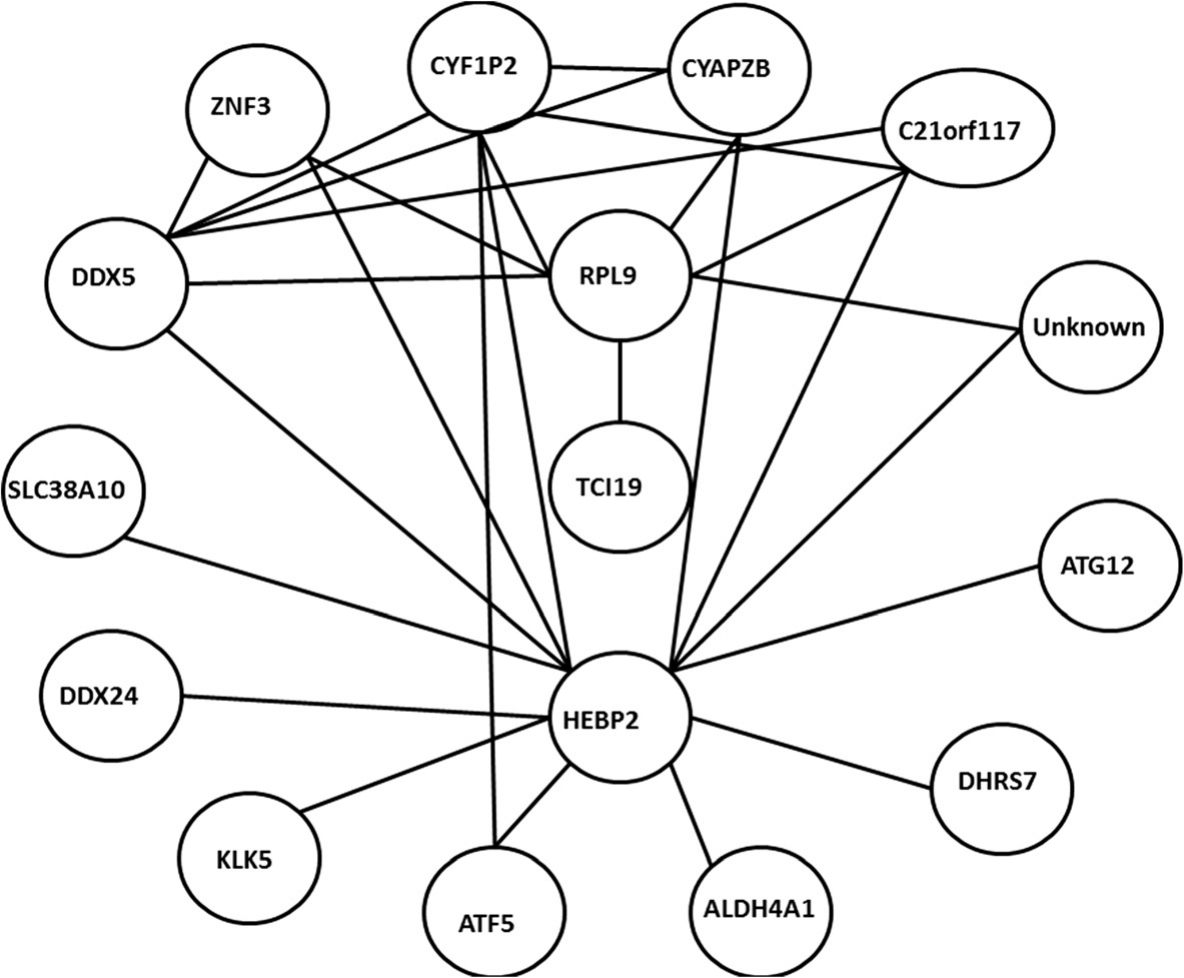
A target network of 52 genes selected by AMFES for GSE4227.

The clustergram function of the genes selected from the GSE4226 dataset is described as an example to support the target networks constructed. Only the top ranked 15 genes are used for the analysis shown in Figure 6. In fact, any number of genes can be selected to follow the same procedure. The complete target network constructed by 74 biomarkers for GSE4226 and 52 biomarkers for GSE4227 are shown in Figures 7 and 8, respectively. Because of the limited visibility of the figure constructed by a large number of biomarkers for GSE4229, we did not include it in the manuscript. For the target networks constructed, we observed that only a few of the interactions between biomarkers are reliable after trimming by a permutation test. This observation can help focus on a smaller set of the more important interactions.

**Figure 9:**
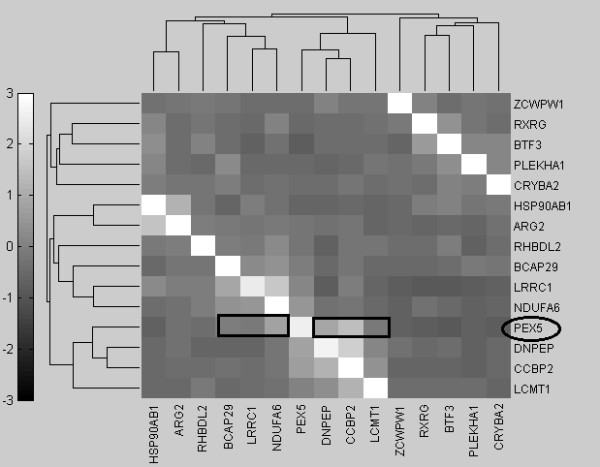
Clustergram of first 15 genes selected by AMFES for GSE4226.

**Figure 10:**
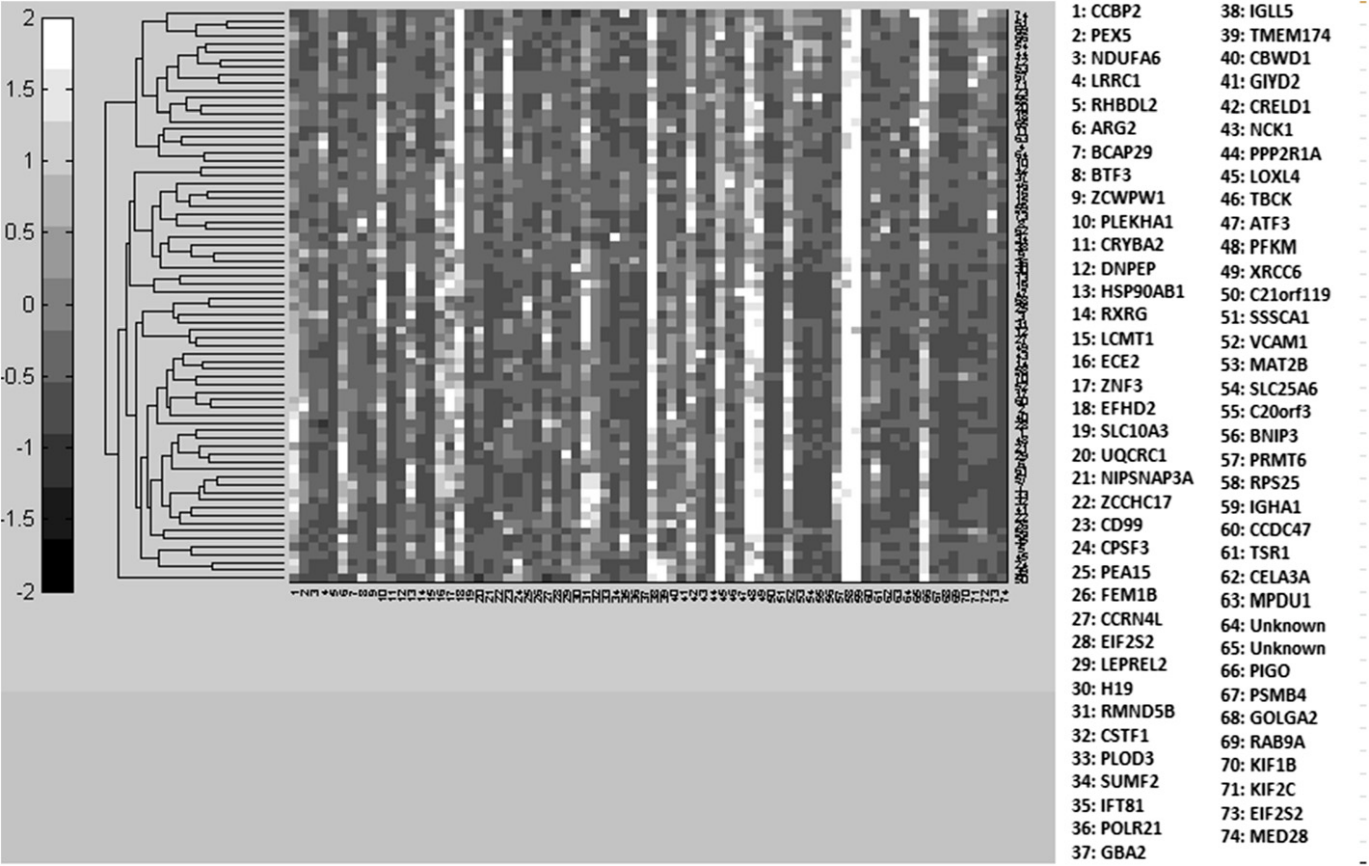
Clustergram of 74 genes selected by AMFES for GSE4226.

**Figure 11:**
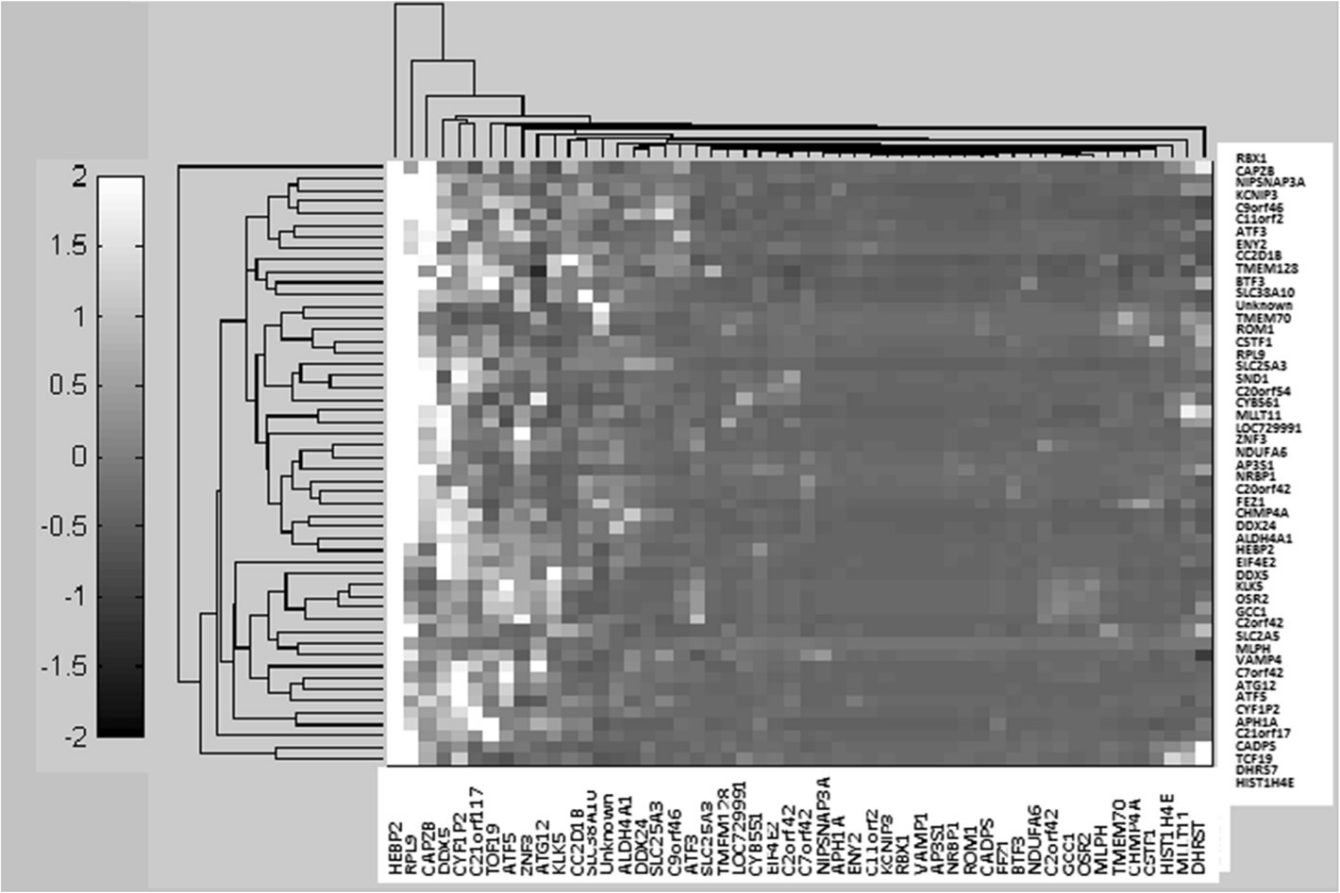
Clustergram of 52 genes selected by AMFES for GSE4227.

As the heatmap generated for the 15 genes (Figure 9) shows, if a few genes share high pairwise MI values with a specific gene, they tend to cluster together as indicated by rectangles and have fewer “hops” (number of connections between a pair of gene) than other genes (Figure 6). For example, PEX5 shares similar MI values with DNPEP, CCBP2, LCMT1, BCAP29, LRRC1 and NDUFA6, which are clustered together in Figure 9. From the graphical view of the target network, these genes have direct connections to PEX5, as shown in Figure 6. On the other hand, a gene such as PLEKHA1 which is two hops away from PEX5 displays an obvious color difference in the MI clustergram. The clustered heatmaps of 74 genes for GSE4226 and of 52 genes for GSE4227 are shown in Figures 10 and 11, respectively.

### Gender analysis

AMFES is used to analyze the data by gender for all three datasets and the results are shown in Table 4. The complete lists of selected female and male genes for GSE4226 are shown in Tables 5 and 6 as examples, respectively. The complete lists of selected female and male genes for GSE4227 and GSE4229 are shown in Additional file 13, Additional file 14, Additional file 15, and Additional file 16. Interestingly, from all the results there are no overlapped biomarkers between female and male genes.

**Table 4:** The comparisons of female genes and male gene selected by AMFES

**Datasets**	**Number of features selected for female**	**Number of features selected for male**
GSE4226	19	9
GSE4227	12	13
GSE4229	36	13

**Table 5:** The selected female genes of GSE4226

**Number**	**Gene symbols**
1	Unknown ID
2	SKIP
3	Unknown ID
4	MGC15504
5	DKFZP434J046
6	FLJ20591
7	ELA3B
8	CADPS
9	FLJ10707
10	FLJ14639
11	ZDHHC1
12	Unknown ID
13	LOXL4
14	PTPN4
15	STK25
16	CNP
17	TERF21P
18	TAF12
19	BAP29

**Table 6:** The selected male genes of GSE4226

**Number**	**Gene symbols**
1	RNASE1
2	FLJ22729
3	FLJ12571
4	IDH2
5	HCS
6	E1B-AP5
7	PIGO
8	ZDHHC3
9	H4FJ

## Discussion

In this paper, the GSE 4226 dataset is studied in more detail because the number of female and male subjects is equal, thereby avoiding the biased sampling problem of the datasets (i.e., when the number of samples is unbalanced for two classes). Traditionally, statistical software such as SAM (Significance Analysis of Microarrays) [27], PAM (Prediction Analysis for Microarrays) [28] or ANOVA (Analysis of Variance) are used for analyses of biomarkers [12-14]. Compared to the results in [12-14], AMFES selects a much smaller, yet important, set of biomarkers which are supported by the cross-validation. In [7,8,29], the researches were performed based on SVM-RFE for AD biomarker analyses. Here, AMFES can appreciably improve the performance of biomarker analysis. In our current research, we are extending the framework of Zhang et al. [6] by AMFES, and this work will be reported shortly. Finally, interestingly for the gender analysis, when we compare results for female AD subjects with those for male AD subjects, there are no overlapping genes, indicating that the important biomarkers may differ according to gender.

## Conclusions

**Figure 12:**
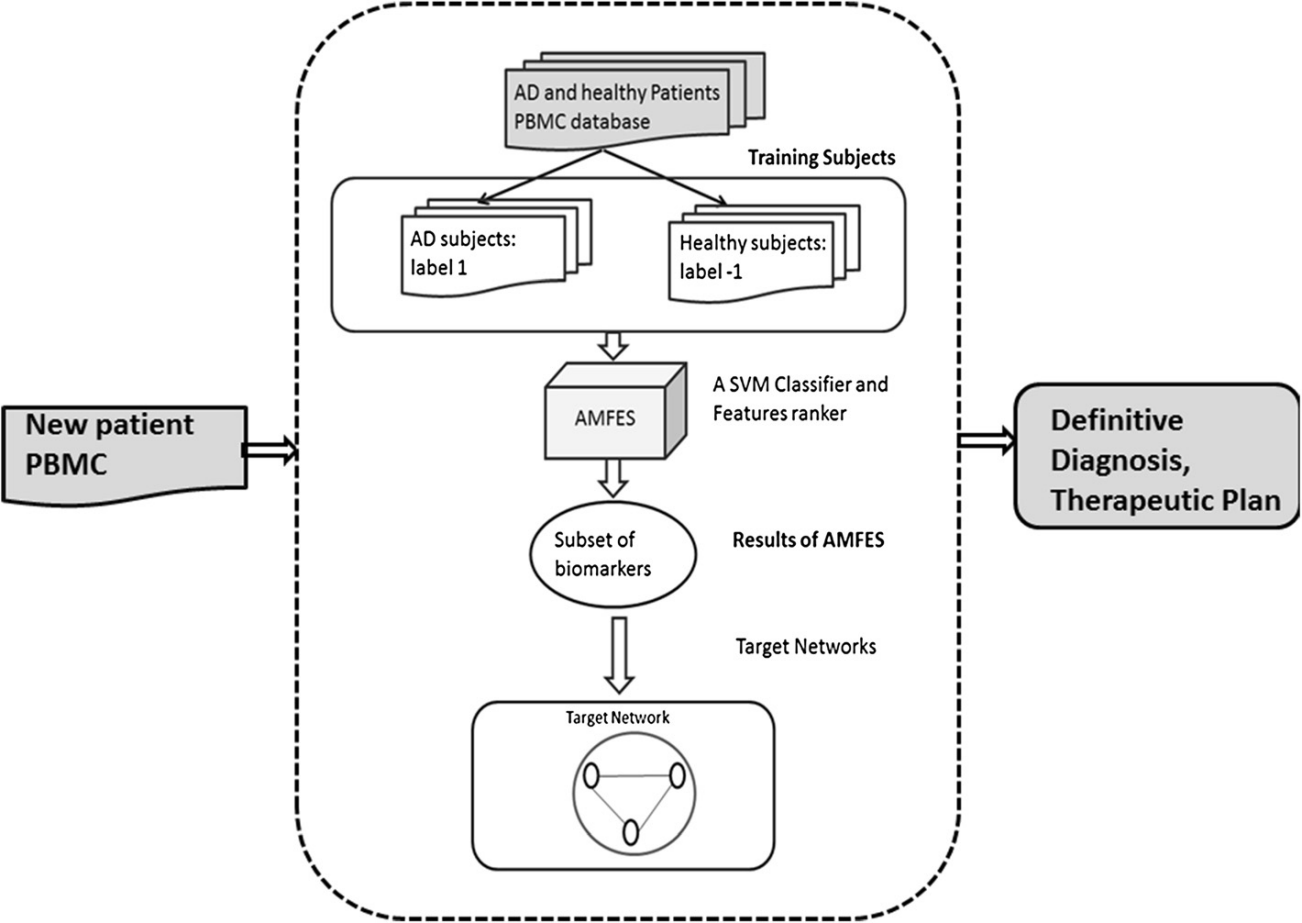
A complete process to improve diagnosis of AD by AMFES.

Based on above results, we have proposed a methodology for improving the diagnosis of AD, which is summarized in the Figure 12. As shown in Figure 12, after labeling the AD vs. normal samples, AMFES can select a small subset of important biomarkers by evaluating them on an SVM. For a new patient, the proposed method can select the biomarkers accordingly and construct the corresponding target networks to provide a definitive diagnosis. As in [11], the target networks can be used for further development of a synergistic strategy to improve the therapy of AD in the future.

## Competing interests

The authors declare that they have no competing interests.

## Authors’ contributions

Please see sample text in the instructions for authors. WH, CD: Implementation of project. SC: Design the project. All authors read and approved the final manuscript.

## Supplementary Material

Additional file 1 **GSE4226_RawData_Normalized.xlsx.** An MS Office Excel file which contains normalized raw data of GSE4226 samples. Click here for file

Additional file 2 **GSE4227_RawData_Normalized.xlsx.** An MS Office Excel file which contains normalized raw data of GSE4227 samples. Click here for file

Additional file 3 **GSE4229_RawData_Normalized.xlsx.** An MS Office Excel file which contains normalized raw data of GSE4229 samples. Click here for file

Additional file 4 **GSE4226_74_Biomarkers.xlsx.** An MS Office Excel file which contains a list of gene symbols of 74 biomarkers of GSE4226 samples. Click here for file

Additional file 5 **GSE4227_52_Biomarkers.xlsx.** An MS Office Excel file which contains a list of gene symbols of 52 biomarkers of GSE4227 samples. Click here for file

Additional file 6 **GSE4229_395_Biomarkers.xlsx.** An MS Office Excel file which contains a list of gene symbols of 395 biomarkers of GSE4229 samples. Click here for file

Additional file 7 **GSE4226 AD MI.xlsx.** An MS Office Excel file which contains a matrix of the pairwise MI values of 74 biomarkers of AD samples. Click here for file

Additional file 8 **GSE4226 Normal MI.xlsx.** An MS Office Excel file which contains a matrix of the pairwise MI values of 74 biomarkers of normal samples. Click here for file

Additional file 9 **GSE4227 AD MI.xlsx.** An MS Office Excel file which contains a matrix of the pairwise MI values of 52 biomarkers of AD samples. Click here for file

Additional file 10 **GSE4227 Normal MI.xlsx.** An MS Office Excel file which contains a matrix of the pairwise MI values of 52 biomarkers of normal samples. Click here for file

Additional file 11 **GSE4229 AD MI.xlsx.** An MS Office Excel file which contains a matrix of the pairwise MI values of 395 biomarkers of AD samples. Click here for file

Additional file 12 **GSE4229 Normal MI.xlsx.** An MS Office Excel file which contains a matrix of the pairwise MI values of 395 biomarkers of normal samples. Click here for file

Additional file 13 **GSE4227 Female Gene List.xlsx. **An MS Office Excel file which contains a complete list of gene symbols of female genes selected for GSE4227. Click here for file

Additional file 14 **GSE4227 Male Gene List.xlsx.** An MS Office Excel file which contains a complete list of gene symbols of male genes selected for GSE4227. Click here for file

Additional file 15 **GSE4229 Female Gene List.xlsx.** An MS Office Excel file which contains a complete list of gene symbols of female genes selected for GSE4229. Click here for file

Additional file 16 **GSE4229 Male Gene List.xlsx.** An MS Office Excel file which contains a complete list of gene symbols of female genes selected for GSE4229. Click here for file
